# State-of-the-Art 2D and Carbon Nanomaterials in France

**DOI:** 10.3390/nano13212826

**Published:** 2023-10-25

**Authors:** Catherine Journet

**Affiliations:** Laboratoire des Multimatériaux et Interfaces (LMI-UMR 5615 CNRS / UCBL), Domaine Scientifique de la Doua, Université Claude Bernard Lyon 1, Bâtiment Chevreul, 6 rue Victor Grignard, CEDEX, 69622 Villeurbanne, France; catherine.journet-gautier@univ-lyon1.fr

Nanotechnology has revolutionized various industries by enabling the manipulation and fabrication of materials at the nanoscale. In the realm of nanomaterials, 2D materials and carbon nanomaterials have emerged as frontrunners, promising a plethora of applications ranging from electronics to energy storage. France, with its rich scientific heritage, has been at the forefront of nanomaterials research, and the journal *Nanomaterials* proudly presents a Special Issue titled “State-of-the-Art 2D and Carbon Nanomaterials in France”, showcasing the groundbreaking research from some of the most eminent researchers in the field. The various publications cover a wide range of topics, including the synthesis, characterization, and applications of various carbon-based nanomaterials. These contributions shed light on the significant progress made in this area and collectively offer valuable insights into the current state and prospects of nanomaterial research in France.

One of the key areas of focus in this issue concerns the synthesis of vertically aligned carbon nanotubes (VACNTs), wherein researchers in France have made noteworthy contributions and their insights have the potential to revolutionize the applications and production of VACNTs. By exploring various factors such as carbon source content [1], Fe/C ratio [1], catalyst feeding [2], and conducting interfaces [3], they have provided valuable information for optimizing the synthesis process, resulting in tailored properties of carbon nanotubes suitable for specific applications. The implications of their findings are far-reaching, enabling scalable and controlled production of VACNTs, thereby unlocking numerous avenues for widespread commercial utilization. This groundbreaking achievement is poised to be a game-changer, bringing us closer to harnessing the full potential of VACNTs for large-scale industrial use.

Furthermore, within the realm of carbon nanomaterials, this issue presents cutting-edge research exploring the world of curved polyaromatic hydrocarbons, known as carbon nanobelts. Employing laser-induced vaporization and ionization techniques, scientists have investigated collision impacts between species, leading to mass loss and the formation of new circular strained dehydrobenzoannulene species [4]. These findings not only provide important information on collision degradation routes of curved molecular carbon species, but also offer insights into the high-energy impact conditions observed in certain astrochemical environments.

Another exciting contribution to this issue is the exploration of germanane, a two-dimensional material with stacks of atomically thin germanium sheets. The study focused on improving germanane’s electrical transport properties to develop atomic-scale devices [5]. The researchers investigated the electrical transport of annealed methyl-terminated germanane microcrystallites, revealing promising electrical properties suitable for complementary metal oxide semiconductor back-end-of-line processes. This study shed light on the challenges and promise of germanane as a two-dimensional material and brings it closer to practical application in electronic devices, enriching the realm of nanomaterial-based technologies.

The potential of hetero-bilayer structures, where strain and spin-orbit coupling can be engineered, is also explored in this Special Issue, revealing exciting possibilities for tuning electronic band structures in van der Waals materials. Scientists have investigated the band structure of the WS_2_/graphene hetero-bilayer by varying the twist angle [6]. Their work demonstrated that strain quantitatively affects electronic features of the WS_2_ monolayers, including the tunable WS_2_ spin-orbit splitting, offering new avenues for controlling the band dispersion of van der Waals materials. The insights gained from this research offer exciting opportunities for designing novel electronic devices and uncovering previously unexplored physical phenomena, holding immense potential for the electronics industry and future technological advancements.

Additionally, this issue includes research on the transfer process of monocrystalline graphene and its influence on the quality, morphology, and electrical properties of the transferred graphene [7]. The controlled bubbling electrochemical delamination transfer technique was identified as a suitable method for transferring large single-crystal graphene without degrading its quality, offering valuable insights for its practical applications and paving the way for the development of reliable high-performance graphene-based devices.

Moreover, this Special Issue delves into the activation of graphene oxide, using CO_2_ and KOH-based approaches, to enhance CO_2_ adsorption capacity [8]. These activation methods significantly increased the specific surface area and CO_2_ adsorption performance, showcasing the potential for efficient CO_2_ capture using activated graphene. This research highlights the significance of activation techniques in enhancing the surface and porosity of carbon nanomaterials, opening up potential carbon dioxide capture applications. Such advancements are critical in addressing environmental challenges and exploring sustainable energy solutions.

In addition, the exploration of hydrogen storage in nanoporous carbons holds promise for unlocking the potential of hydrogen-based energy systems. A review in this Special Issue explores the possibilities of nanoporous carbons for effective hydrogen storage by physisorption, highlights the current limitations, and proposes strategies to improve storage performance, including chemical modifications of carbon pore walls [9]. Preliminary results of boron-substituted nanoporous carbons have demonstrated promising improvements in hydrogen adsorption energy, encouraging further research in this field. Understanding the behavior and properties of these materials is indeed essential for realizing their potential in hydrogen-based energy scenarios.

Finally, the complex structure and extraordinary properties of glass-like carbon (GLC), a vitreous material with exceptional properties that remain relatively unexplored, are discussed. By reviewing different models and a physical route involving pulsed laser deposition, the author outlines our understanding of GLC structure, topology, and geometry [10]. The analysis also extends to 2D materials, offering a comprehensive approach to exploring GLC and other similar materials. This work sets the stage for further research into harnessing the unique properties of vitreous carbon in various fields, from electronics to aerospace applications.

To summarize, the contributions in this issue cover a wide range of topics, including the design of novel materials, the development of new synthesis methods, and the characterization of the properties of nanomaterials. French researchers have been at the forefront of advancing the understanding of carbon-based nanomaterials, such as carbon nanotubes and fullerenes, exploring their unique electronic, optical, and mechanical properties.

One of the most exciting aspects of the research highlighted in this issue is its potential for real-world impact. For example, the development of 2D materials with tailored properties could lead to breakthroughs in electronics and photonics, while the use of carbon-based nanomaterials in energy storage devices could help to address the growing demand for renewable energy. Additionally, the biocompatibility of these materials makes them promising candidates for use in biomedical applications such as drug delivery and biosensing.

Overall, this Special Issue of *Nanomaterials* provides a comprehensive and up-to-date overview of the latest research in 2D and carbon-based nanomaterials in France. The contributions of French researchers in this field are significant, and their work is helping to advance our understanding of the unique properties and potential applications of these materials. As nanotechnology continues to evolve, it is clear that France will remain a key player in this exciting field. As we move forward, the collaboration between various stakeholders will be vital in translating these discoveries into practical solutions that benefit society and contribute to a more sustainable future.

We hope that this collection will provide a comprehensive overview of the current state-of-the-art of 2D and carbon nanomaterials research in France. We would like to express our gratitude to the authors for their valuable contributions to this Special Issue. We also thank the reviewers for their insightful comments and suggestions, which have greatly improved the quality of the papers. Finally, we hope that this Special Issue will inspire further research and collaboration in the field of nanomaterials in France and beyond.
